# Cryopreservation Protocol Optimization for *Penaeus monodon* Sperm: Reagent Screening and Parameter Refinement

**DOI:** 10.3390/biology14040408

**Published:** 2025-04-11

**Authors:** Dewei Kong, Song Jiang, Jianzhi Shi, Qibin Yang, Jianhua Huang, Yundong Li, Yangyang Ding, Jieyi Wang, Xinyu Qi, Tianmi Liu, Falin Zhou

**Affiliations:** 1 South China Sea Fisheries Research Institute, Chinese Academy of Fishery Sciences, Key Laboratory of South China Sea Fishery Resources Exploitation and Utilization, Ministry of Agriculture and Rural Affairs, Guangzhou 510300, China; kdw0204@163.com (D.K.); tojiangsong@163.com (S.J.); shijianzhi1989@163.com (J.S.); yangqibin1208@163.com (Q.Y.); hjh210440@sina.com.cn (J.H.); liyd2019@163.com (Y.L.); gaoguimao@gmail.com (Y.D.); 15020485966@163.com (J.W.); qjgqxy@163.com (X.Q.); 2 State Key Laboratory of Mariculture Biobreeding and Sustainable Goods, Qingdao 266071, China; 3 Fisheries Technology Extension Center of Hainan Province, Haikou 570311, China

**Keywords:** *Penaeus monodon*, cryopreservation, sperm, ultrastructure, cryoinjury mechanisms

## Abstract

*Penaeus monodon* (black tiger shrimp) is one of the important shrimp species in aquaculture. Cryopreserving its sperm not only provides technical support for breeding but also effectively prevents the decline of genetic resources, promoting the sustainable development of its aquaculture industry. This study screened different types of diluents, cryoprotectants, and concentrations and explored equilibration time, cooling protocols, and thawing conditions, ultimately determining the optimal cryopreservation protocol for *P. monodon* sperm. The results showed that the optimal cryopreservation protocol involved using natural seawater as the diluent with 10% dimethyl sulfoxide (DMSO) as the cryoprotectant, in which the sperm suspension and cryoprotectant were mixed at a 1:1 (*v*/*v*) ratio and equilibrated at 4 °C for 30 min. Subsequently, cooling was performed using a programmable controlled-rate freezer: the temperature was reduced to −20 °C at −5 °C/min and held for 5 min; then cooled to −80 °C at −10 °C/min and held for 5 min; finally, the temperature was reduced to −180 °C at −20 °C/min. After cooling, the sperm samples were transferred to liquid nitrogen for long-term storage. The results demonstrated that thawing in a 37 °C water bath achieved the highest sperm motility compared to conditions at 27 °C, 32 °C, 42 °C, and 60 °C. After 15 days of liquid nitrogen storage, the sperm survival rate was 53.33 ± 9.18%. Scanning electron microscopy (SEM) and transmission electron microscopy (TEM) observations revealed that the sperm structure was intact before freezing, with a rounded head, a distinct acrosomal spike anterior to the head, a concentrated nucleus in the head, dense chromatin, and a smooth cell membrane surface. However, after freezing and thawing, the acrosomal spikes of some sperm were fractured, and the membrane structure was damaged. Enzyme activity analysis showed that during liquid nitrogen storage from 0 to 15 days, the enzyme activity of alkaline phosphatase (AKP) and acid phosphatase (ACP) in sperm gradually increased with significant differences observed compared to day 0 (*p* < 0.05). The activity of malondialdehyde (MDA) showed a gradual increase at 0, 5, and 10 days, but then decreased at day 15. The enzyme activity of catalase (CAT) showed no significant changes from 0 to 10 days (*p* > 0.05) but significantly increased on day 15 (*p* < 0.05). The activity of total superoxide dismutase (T-SOD) showed no significant changes from 0 to 5 days (*p* > 0.05) but significantly increased from days 10 to 15 (*p* < 0.05). These findings provide valuable insights into the cryopreservation of *P. monodon* sperm and will guide the optimization of cryoprotectant combinations and freezing protocols aimed at improving sperm survival rates.

## Simple Summary

This study addresses critical issues in the cryopreservation protocol for *Penaeus monodon* shrimp sperm by proposing an optimized long-term preservation strategy. While previous studies validated the efficacy of 5% DMSO in sperm cryopreservation and established corresponding protocols, they lacked systematic analysis of ultrastructural integrity, enzymatic activity, and long-term preservation outcomes. To bridge these gaps, we present three innovations: First, the cryoprotectant formulation was optimized through the integration of 10% DMSO with natural seawater as a biocompatible carrier. Second, programmable controlled-rate freezing technology was implemented to minimize cryodamage during the freezing process. Third, post-thaw sperm quality was comprehensively evaluated via ultrastructural microscopy (SEM/TEM) coupled with metabolic activity profiling. After 15 days of cryopreservation, our protocol achieved 85% sperm viability with only minor acrosomal membrane damage, outperforming conventional methods in functional preservation. These advancements not only extend the effective cryopreservation window but also elucidate mechanisms underlying cryodamage mitigation, addressing critical gaps in crustacean reproductive biotechnology. By enabling reliable preservation of genetic resources, this work provides a technical foundation for sustainable aquaculture and shrimp germplasm bank development.

## 1. Introduction

*Penaeus monodon* (black tiger shrimp) holds significant economic importance in China’s mariculture sector [1]. Remarkable advancements have been achieved and implemented in the full artificial breeding technology of *P. monodon*, both in production practices and research endeavors. The widespread promotion of new, artificially bred varieties of *P. monodon* has catalyzed the rapid growth of the industry. However, in practical production and research scenarios, the effective utilization period of male shrimp is relatively brief, generally confined to the reproductive season. Consequently, the long-term preservation of reproductive cells from high-quality germplasm resources has emerged as a pressing scientific challenge within the *P. monodon* industry. Furthermore, for the conservation of germplasm resources and the development of novel germplasm, it is imperative to ensure the prolonged preservation of reproductive cells to optimize resource utilization.

Cryopreservation technology, as a reliable method, plays an important role in aquaculture [2]. Sperm cryopreservation technology not only allows long-term storage of sperm, preserving its desirable traits, but also helps avoid issues related to in vivo preservation caused by factors such as gene loss, generation gaps, or genetic drift [3]. This technology significantly reduces breeding costs, addresses issues of geographic isolation and asynchronous development of male and female gametes, and provides convenience for genetic breeding research. In addition, cryopreservation technology can also minimize the impact of external environmental factors on germplasm resources, prevent the spread of animal-derived diseases, provide high-quality materials for artificial breeding and the creation of new germplasm, expand the selection of excellent trait populations, and open up new ways to establish a germplasm resource bank [4,5].

To date, research on fish sperm cryopreservation has matured, with successful cryopreservation achieved in at least 200 species of fish [6,7], providing significant value for the preservation of fish germplasm resources and genetic breeding research. Furthermore, a reliable procedure for cryopreservation of sperms has been documented in decapod crustaceans. Examples include *Sicyonia ingentis* [8], *Macrobrachium rosenbergii* [9,10,11], *Fenneropenaeus indicus* [12], *Penaeus chinensis* [13], *Penaeus monodon* [14,15], and *Litopenaeus vannamei* [16].

The cryopreservation technology for *P. monodon* sperm has gradually developed into a defined research framework. Early studies primarily focused on screening cryoprotectant types and concentrations. Vuthiphandchai et al. [15] first established a cryopreservation protocol using 5% dimethyl sulfoxide (DMSO), demonstrating its efficacy in maintaining sperm viability for up to 60 days. However, this study did not explore cryoinjury mechanisms or long-term preservation effects (>90 days). Subsequent research highlighted distinctions between spermatophore-based and individual sperm cryopreservation. Bart et al. [14] validated the effectiveness of 5% DMSO in spermatophore cryopreservation, proposing that the structural integrity of spermatophores may enhance sperm survival by providing physical protection. Nevertheless, the advantages of spermatophore preservation may obscure unique challenges inherent to individual sperm cryopreservation, such as variability in osmotic tolerance and increased membrane fragility. Consequently, protocol optimization for single-sperm cryopreservation requires independent investigation to address these specific cryobiological constraints.

Recent advancements have expanded cryoprotectant selection. Rosmiati et al. [17] systematically compared the cryoprotective efficacy of DMSO, methanol, and glycerol on *P. monodon* sperm, identifying 10% glycerol as superior for maintaining sperm viability in liquid nitrogen storage. However, this evaluation was limited to short-term effects within 30 days. Grace and Natarajan [18] reported enhanced protection of sperm motility using 10% DMSO compared to lower concentrations, yet failed to assess potential structural damage caused by elevated cryoprotectant concentrations. These findings underscore a trade-off between the cryoprotectant concentration and cryoinjury severity. Current research remains constrained by short-term evaluations of single cryoprotectants, lacking comprehensive analyses of combined cryoprotectants, freeze–thaw protocols, and cellular damage mechanisms.

Although existing studies have identified critical parameters for protocol optimization (e.g., cryoprotectant selection, extender composition, cooling rates), technical translation remains hindered by systemic limitations. As noted by Aquino et al. [19] in their review, key challenges in crustacean sperm cryopreservation include membrane lipid sensitivity to hypothermic stress and the absence of standardized damage assessment metrics. Current methodologies predominantly rely on superficial indicators, such as survival rates, while quantitative analyses of mitochondrial function, membrane integrity, and enzymatic activities (e.g., ATPase, antioxidant enzymes) remain underdeveloped. This knowledge gap impedes the mechanistic understanding of cryoinjury and consequently restricts precision optimization of cryoprotectant formulations.

Although previous studies have investigated cryoprotectants and their concentrations for sperm cryopreservation in *P. monodon*, further optimization remains necessary. This study aimed to improve sperm survival rates and biological activity during long-term cryopreservation by refining cryoprotectant formulations, diluent compositions, and freeze–thaw protocols. Ultrastructural analysis and enzyme activity assays were integrated to elucidate cellular damage mechanisms during cryopreservation, thereby providing a theoretical foundation for protocol development. The findings are expected to advance reproductive regulation, selective breeding of superior varieties, and sustainable aquaculture practices for *P. monodon*.

## 2. Materials and Methods

### 2.1. Broodstock Management

The *P. monodon* used in the experiment was sourced from the Shenzhen Experimental Base of the South China Sea Fisheries Research Institute, Chinese Academy of Fishery Sciences. Prior to the experiment, the shrimp were cultured in filtered, aerated seawater at 28 ± 1 °C, with a pH of 7.8–8.2 and a salinity of 30–33 ppt, and were fed commercial shrimp formula feed (produced by Dongteng Feed Company, Zhanjiang, China). The main nutritional components of this feed include: crude protein 41.0%, crude fat 4.0%, crude fiber 5.0%, crude ash 16.0%, moisture 12.0%, calcium 2.20%, phosphorus 4.00%, and it is rich in various vitamins, such as vitamin A, D3, E, B1, B2, and B6. They were fed three times per day, with each feeding amounting to 3–5% of body weight. The sperm bundle collection of *P. monodon* was carried out using the squeezing method [20]. Thirty sexually mature male shrimp were selected, and all tools (collection tubes, pipettes, etc.) were sterilized at high temperatures or treated with 70% ethanol before the experiment. The entire operation was completed in a clean laboratory, with work surfaces and equipment disinfected using ultraviolet light, and the operators wore sterile gloves and masks. The specific procedure was as follows: the shrimp body was rinsed with sterile seawater, and the development of the testes at the base of the fifth pereiopod was visually inspected (white opaque masses were the determining indicator). A 5% iodine solution or potassium permanganate solution was used to disinfect the genital pore surface. The sperm bundle waas expelled by applying directional pressure with the thumb and index finger on the base of the fifth pereiopod, and the sample was immediately collected with sterile tweezers to avoid contamination.

### 2.2. Sperm Collection

Fresh sperm bundles of *P. monodon* were dried with sterile filter paper to remove surface moisture (the processing time was strictly controlled to within 2 min to prevent sperm vitality decline due to exposure to room temperature) and then weighed and transferred to a 400-mesh gauze screen. The gauze screen was immersed in a beaker containing 4 °C pre-treated seawater (filtered through sand and disinfected by UV), and sperm were quickly squeezed out of the sperm bundles using sterile tweezers. The entire process was carried out under ice bath conditions (with sperm bundle sample temperature maintained at 4 °C to ensure cell viability, and the operation time not exceeding 3 min). The resulting homogenate was transferred to a 2 mL centrifuge tube and purified by gradient centrifugation: the first stage was centrifugation at 500× *g* for 10 min to remove seminal plasma proteins and cell debris; the second stage was centrifugation at 2000× *g* for 8 min to enrich intact sperm cells. The centrifugation parameters were optimized based on the cell sedimentation curve analysis from pre-experiments.

### 2.3. Sperm Viability Assessment

Sperm vitality was assessed using the trypan blue staining method [7,12,21,22] to quantitatively measure the survival rate (*n* = 30), using a hemocytometer (25 × 16 small squares, 0.1 mm^3^) for detection. Before the experiment, the sperm suspension was diluted to 10^7^ sperm/mL and mixed with trypan blue staining solution at a 1:1 ratio, followed by a 3-min staining period before microscopic observation. Viable sperm remained unstained due to their intact structure, while dead sperm appeared blue. For each sample, 500 sperm were randomly counted, and only samples with a survival rate ≥85% were used for further study. All experiments were repeated independently three times and performed using the trypan blue staining cell viability detection kit (Beyotime, C0011, Hainan Luhengyuan Biological Technology Co., Ltd., Haikou, China) to ensure data reliability and experimental stability.

### 2.4. Screening of Cryopreservation Conditions for P. monodon

#### 2.4.1. Determination of Suitable Diluents

Based on preliminary screening, this study selected natural seawater (collected from the coastal waters of Dapeng Peninsula, Shenzhen, China, filtered through a 0.45 μm membrane and sterilized at 121 °C under high pressure), Ringer’s solution, Hanks’ balanced salt solution (HBSS), and D-Hanks balanced salt solution (all purchased from Shantou Xilong Scientific Co., Ltd., Shantou, China, analytical grade with purity ≥ 99%) as the basic diluents. All solutions were independently tested three times for osmolarity using an osmometer (Gonotec Osmomat 3000): natural seawater had an osmolarity of 1020 ± 15 mOsm/L, Ringer’s solution was 305 ± 3 mOsm/L (containing Ca^2+^ 2.4 mM, K^+^ 5.0 mM), Hanks’ balanced salt solution was 290 ± 2 mOsm/L, and D-Hanks balanced salt solution was 295 ± 4 mOsm/L. Before use, all solutions were calibrated with a pH meter (Mettler Toledo FE28) to pH 7.8 ± 0.1 and stored in the dark at 4 °C for no more than 72 h to ensure the stability of their physicochemical properties. Dimethyl sulfoxide (DMSO, from the same brand) was selected as the cryoprotectant. DMSO plays a key protective role in sperm cryopreservation by inhibiting ice crystal formation and balancing the osmotic pressure inside and outside the cells. The cryoprotectant solution was prepared by mixing DMSO with the four diluents at final concentration gradients of 5%, 10%, and 15% (for example, the 5% concentration group was mixed at a DMSO: diluent = 1:19 volume ratio). The sperm suspension (500 μL) was mixed with an equal volume of cryoprotectant (1:1 ratio) on ice, and the mixture was left to stand for 30 min to achieve osmotic equilibrium. A three-phase cooling program was then applied: first, cooling at a rate of −5 °C/min to −20 °C and holding for 5 min; second, cooling at −10 °C/min to −80 °C and holding for 5 min; finally, cooling at −20 °C/min to −180 °C before immersion in liquid nitrogen for long-term storage. During thawing, the samples were rapidly rewarmed in a 37 °C water bath for 2 min, and three biological replicates were set for each experimental group.

#### 2.4.2. Determination of Suitable Cryoprotectants

Methanol, DMSO, ethylene glycol, 1,2-propanediol, and glycerol were chosen as cryoprotectants, and concentrations of 5%, 10%, and 15% were prepared. Natural seawater served as the diluent, and the sperm was mixed with the cryoprotectant in a 1:1 ratio. The cooling process was carried out as previously described, and the samples were then stored in liquid nitrogen. After one hour, the samples were thawed, and sperm viability was measured. This experiment was repeated three times for accuracy.

#### 2.4.3. Different Equilibration Times at 4 °C

Using natural seawater as the diluent, sperm was mixed with a 10% DMSO cryoprotectant in a 1:1 ratio and then allowed to stand at 4 °C for various durations: 0, 10, 20, 30, and 40 min. Following the cooling protocol (P-2), the samples were stored in liquid nitrogen. After one hour, the samples were thawed, and sperm viability was measured. This experiment was repeated three times to ensure accuracy.

#### 2.4.4. Development of Cryopreservation Protocol

Using natural seawater as the diluent, sperm was mixed with a 10% DMSO cryoprotectant in a 1:1 ratio and allowed to stand at 4 °C for 30 min. Various cooling protocols were then applied to the samples, which were subsequently stored in liquid nitrogen for one hour. After this period, the samples were thawed in a 37 °C water bath, and sperm viability was measured. This experiment was repeated three times to ensure accuracy.

The experiment utilized a programmed cooling device (Model CJL37, produced by Chengdu Haier Biomedical Science Co., Ltd., Chengdu, China) and a liquid nitrogen tank (Model YDZ-100, also produced by Chengdu Haier Biomedical Science Co., Ltd., Chengdu, China) for programmed cooling of experimental samples, configuring four different freezing cooling protocols (Table 1). The chamber of the cooling device was dried for 30 min to ensure it remained dry, and the cooling program was set to maintain equilibration at 4 °C before placing the sperm samples from the four treatment groups inside. After cooling the sperm samples to the set temperature using the programmed cooling method, they were quickly transferred to the liquid nitrogen tank. The samples were thawed in a 37 °C water bath, and three parallel samples from the four treatment groups were placed in the water bath and gently stirred for uniform thawing for 2 min before measuring the survival rate. Three biological replicates were performed for each experimental group.

To systematically investigate the effects of different cooling pathways on sperm cryopreservation efficacy, this study established a multi-stage gradient cooling protocol from 4 °C to −180 °C, comprising four distinct programs (P-1 to P-4). Compared with the conventional two-step cooling method (slow cooling to a specific temperature followed by direct immersion in liquid nitrogen), this protocol introduces additional cooling phases and rate variations to optimize cellular physiological adaptation during phase transitions and mitigate cryoinjury.

Program P-1 served as the baseline reference, employing a single cooling rate (−5 °C/min) to −80 °C before deep cryopreservation. Programs P-2 and P-3 incorporated a 5-min equilibrium plateau at −20 °C, followed by subsequent cooling at −10 °C/min and −20 °C/min respectively, enabling comparative analysis of intermediate cooling rates on cell membrane stability and ice crystal regulation. Program P-4 represented the extreme control, implementing direct cooling from ambient temperature to −80 °C at −5 °C/min, followed by immediate immersion in liquid nitrogen (−196 °C). All programs included a standardized 5-min equilibration at −80 °C, a parameter derived from preliminary experiments on cryoprotectant permeation kinetics, designed to mitigate osmotic stress from thermal shocks and ensure metabolic arrest.

Notably, all protocols concluded with a rapid cooling phase (−20 °C/min) to traverse the ice crystal formation-sensitive zone (−60 °C to −120 °C), thereby suppressing large ice crystal growth and reducing structural damage. This multi-stage design constitutes a systematic optimization based on prior ice morphology observations, membrane fluidity assessments, and thermodynamic simulations. By fine-tuning gradient nodes, equilibrium durations, and cooling rate combinations, the protocol aims to precisely regulate intracellular/extracellular water transport and ice nucleation processes, ultimately enhancing post-thaw cell viability and integrity. The four-phase cooling strategy seeks to overcome limitations of conventional two-step methods through refined thermal control tactics, theoretically better aligning with cellular physiological demands during dynamic cooling processes.

#### 2.4.5. Determination of Sperm Thawing Temperature

Using natural seawater as the diluent, sperm was mixed with a 10% DMSO cryoprotectant solution in a 1:1 ratio and allowed to stand at 4 °C for 30 min. Following the standard cooling protocol, the samples were stored in liquid nitrogen for 1 h. After this, the samples were thawed in water baths at temperatures of 27 °C, 32 °C, 37 °C, 42 °C, and 60 °C, respectively, and then sperm viability was measured. This entire experiment was repeated three times to ensure accuracy.

#### 2.4.6. Cryopreservation Duration in Liquid Nitrogen

Using natural seawater as the diluent, sperm was mixed with a 10% DMSO cryoprotectant solution in a 1:1 ratio and allowed to stand at 4 °C for 30 min. This cooling protocol adhered to the general procedure, and subsequently, the samples were stored in liquid nitrogen for 15 days. Every 3 days, samples were taken, thawed in a 37 °C water bath, and then sperm viability was measured. This entire experiment was repeated three times to ensure accuracy.

### 2.5. Sperm Morphology and Ultrastructure Analysis

Sperm morphology and ultrastructure analyses were conducted based on the descriptions provided by Ulloa-Rodríguez et al. [23] and Quagio-Grassiottoand Oliveira [24], with some necessary modifications. After the sperm samples frozen in liquid nitrogen were rapidly rewarmed in a 37 °C water bath for 2 min, they were immediately centrifuged at 300× *g* for 5 min to remove the cryoprotectant. Subsequently, the samples were washed three times with pre-chilled 0.1 mol/L PBS (pH = 7.4) at 4 °C to maintain membrane stability, following the same pretreatment procedure as for fresh samples. For scanning electron microscopy (SEM), the sperm-containing supernatant was carefully placed in a clean centrifuge tube and centrifuged at 300× *g* for 5 min to collect the pellet. The sperm were then resuspended in electron microscope fixative (Servicebio, G1102, Wuhan Saiwei Biotechnology Co., Ltd, Wuhan, China), fixed at room temperature for 2 h, and stored at 4 °C. Subsequently, the sperm samples were blocked in 1% OsO_4_ in 0.1 M PBS (pH = 7.4) at room temperature for 1–2 h. After washing three times with PBS (15 min each), the samples were gradually dehydrated in a series of ethanol solutions with increasing concentrations (30%, 50%, 70%, 80%, 90%, 95%, and 100%, twice; 15 min each). The samples were then treated with isoamyl acetate (Sinopharm Chemical Reagent Co., 10003128, Beijing, China) for 15 min and dried using a critical point dryer (Quorum, K850, Quorum Technologies, Newhaven, UK). The dried samples were securely attached to metal stubs using carbon adhesive tabs and coated with gold for 30 s using an ion sputter coater (Hitachi, MC1000, Hitachi High-Technologies Corporation, Tokyo, Japan). Finally, the samples were imaged using a scanning electron microscope (Hitachi, SU8100, Hitachi High-Technologies Corporation, Tokyo, Japan) at Wuhan Saiwei Biotechnology Co., Ltd.

For transmission electron microscopy (TEM), the sperm suspension samples were fixed in electron microscope fixative at room temperature for 1 h and then transferred to fresh TEM fixative in Eppendorf (EP) tubes for further fixation. The samples were washed three times with 0.1 M PBS (pH 7.4) for 15 min each. The sperm samples were then fixed with 1% OsO_4_ in PBS at room temperature for 2 h and washed again with PBS three times (15 min each). The samples were gradually dehydrated in a series of ethanol solutions with increasing concentrations (30%, 50%, 70%, 80%, 95%, and 100%, twice; 20 min each) and in acetone (Sinopharm Chemical Reagent Co., 10000418, Beijing, China) for 15 min. The samples were then infiltrated and embedded in resin using the following steps: a mixture of acetone ands 812 resin (SPI, 90529-77-4, SPI Supplies, West Chester, PA, USA) in a 1:1 ratio was soaked at 37 °C for 2–4 h; a mixture of acetone and 812 resin in a 1:2 ratio was soaked at 37 °C overnight; and pure EMBed 812 was reacted at 37 °C for 5–8 h. After that, pure EMBed 812 was poured into embedding molds, and the sperm were carefully inserted into the resin and stored overnight at 37 °C. The embedding molds with resin and samples were then transferred to a 65 °C oven for polymerization for more than 48 h. The resin blocks were subsequently removed from the molds and stored at room temperature for later use. The resin blocks were sectioned into ultrathin slices (60–80 nm) using an ultramicrotome (Leica, Leica UC7, Leica Microsystems, Wetzlar, Germany), and the sections were floated onto 150-mesh copper grids using Formvar films. The copper grids were stained in the dark with a 2% uranyl acetate saturated ethanol solution for 8 min, washed three times with 70% ethanol, and rinsed three times with ultrapure water. The grids were then stained with 2.6% lead citrate in the dark for 8 min and washed three times with ultrapure water. After drying with filter paper, the copper grids were placed on grid racks and left to dry at room temperature overnight. The grids were observed using a transmission electron microscope (Hitachi, HT7800, Hitachi High-Technologies Corporation, Tokyo, Japan), and the images were captured by Wuhan Sair Biotechnology Co., Ltd., Wuhan, China By comparing the integrity of the plasma membrane, organelle structure, and ice crystal damage characteristics before and after freezing, the study systematically evaluates the effects of different freezing protocols on the ultrastructure of sperm.

### 2.6. Measurement of Antioxidant Enzyme Activity

#### 2.6.1. Sperm Cryopreservation and Pretreatment

Based on the optimal cryopreservation protocol for sperm survival rate obtained through preliminary screening, this study applied the protocol to evaluate the long-term preservation of *P. monodon* sperm samples. The specific procedure was as follows: the sperm suspension was mixed with 10% DMSO cryoprotectant at a 1:1 volume ratio and subjected to programmed gradient cooling. First, the temperature was reduced from 0 °C to −20 °C at a rate of −5 °C/min and held for 5 min to promote cryoprotectant permeation and cell dehydration. Then, the temperature was reduced from −20 °C to −80 °C at a rate of −10 °C/min and held for 5 min. Finally, the temperature was rapidly lowered to −180 °C at −20 °C/min, and the samples were transferred to liquid nitrogen for long-term storage. To evaluate preservation efficacy, thawing tests were performed at 5, 10, and 15 days of storage. The samples were quickly removed from liquid nitrogen and placed in a 37 °C water bath for 2 min of shaking to thaw. Immediately after, they were centrifuged at 300× *g* for 5 min to remove the DMSO-containing supernatant. Then, 0.5 mL of pre-chilled PBS (4 °C, pH 7.4) was added to the sperm pellet and gently resuspended to ensure a cell density ≥10^6^ cells/mL. To fully release intracellular components, the suspension was transferred to a pre-chilled 2 mL glass homogenizer and manually homogenized in an ice–water mixture for 3 min using an intermittent cycle (10 s of homogenization/5 s of pause), until >95% of the cells were broken, as confirmed under the microscope. The homogenate was filtered through a 0.22 μm filter membrane, then aliquoted. Each experimental group was independently repeated 6 times to control operational deviations.

#### 2.6.2. Enzyme Activity Measurement

The activities of several enzymes, including catalase (CAT), total superoxide dismutase (T-SOD), alkaline phosphatase (AKP), and acid phosphatase (ACP), as well as the level of malondialdehyde (MDA), were determined in both fresh and frozen-thawed *P. monodon* sperm samples using enzyme activity assay kits provided by the Nanjing Jiancheng Bioengineering Institute. All procedures were carried out in strict accordance with the manufacturer’s instructions, and each assay was repeated three times to ensure consistency and accuracy. Additionally, each experimental group was replicated six times to further enhance the reliability of the results.

### 2.7. Data Analysis

Statistical analysis was performed using SPSS 21.0 (SPSS Inc., Michigan Avenue, Chicago, IL, USA). A one-way analysis of variance (ANOVA) was used to test for effects. When statistically significant differences were found (*p* < 0.05), Duncan’s multiple range test was used for further comparison of group means. The results are expressed as the mean ± SD (*n* = 3).

## 3. Results

### 3.1. Suitable Diluents

**Figure 1:**
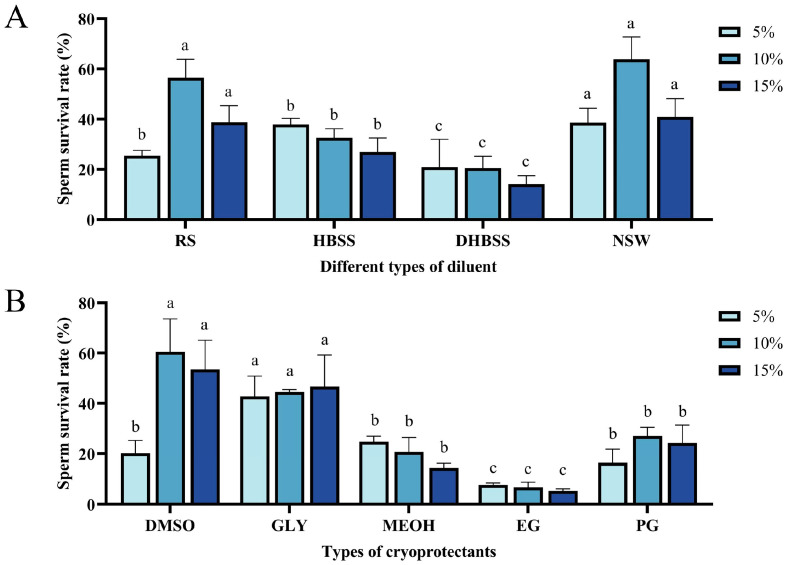
(**A**,**B**) both use two-way ANOVA to show the effects of different DMSO concentrations (5%, 10%, 15%), diluents (RS, HBSS, DHBSS, NSW), and cryoprotectants (glycerol, DMSO, methanol, ethylene glycol, propylene glycol) on the sperm survival rate of *Penaeus monodon* after freezing. The analysis results show that both concentration and the type of diluent/cryoprotectant significantly affect sperm survival rate (*p* < 0.05), and there is a significant interaction between concentration and diluent/cryoprotectant (*p* < 0.05). The letters (a, b, c) in the figure indicate significant differences between different groups (*p* < 0.05). The sample size for all experimental groups was n = 30. The diluents include RS (Ringer’s solution), HBSS (Hanks’ balanced salt solution), DHBSS (DHanks balanced salt solution), and NSW (natural seawater); cryoprotectants include glycerol (Gly), DMSO, methanol (MeOH), ethylene glycol (EG), and propylene glycol (PG).

Two-way ANOVA results showed significant differences in the survival rate of *P. monodon* in different types and concentrations of diluents (*p* < 0.05). Specifically, the type of diluent (natural seawater, Ringer’s solution, D-Hanks solution) and DMSO concentration (5%, 10%, 15%) had a significant effect on the sperm survival rate. Additionally, the interaction between concentration and diluent was also significant (*p* < 0.05). Under the same DMSO concentration, the group with natural seawater as the diluent performed better than the other diluent groups. Particularly, at a 10% DMSO concentration, the natural seawater group had the highest sperm survival rate, reaching 73.08 ± 7.31%, significantly higher than the other diluent groups (*p* < 0.05). The sperm survival rate in the Ringer’s solution (10% DMSO) group was 56.91 ± 6.83%, which performed relatively well. However, the sperm survival rate in the D-Hanks solution group with 15% DMSO was only 16.13 ± 2.12%, the poorest result (Figure 1A). Therefore, natural seawater is considered the most suitable diluent for freezing and preserving *P. monodon* sperm.

### 3.2. Suitable Cryoprotectants

The effect of different cryoprotectants (glycerol, dimethyl sulfoxide DMSO, ethylene glycol, etc.) at various concentrations (5%, 10%, 15%, 20%) was analyzed by two-way ANOVA, which showed that both cryoprotectant type and concentration significantly affected the sperm survival rate (*p* < 0.05). At a concentration of 5%, glycerol showed the best cryoprotectant effect. At 10% and 15% concentrations, dimethyl sulfoxide (DMSO) was more effective than other cryoprotectants. Specifically, at a 10% DMSO concentration, the sperm survival rate reached 60.49 ± 13.14%, significantly higher than that of other cryoprotectants (*p* < 0.05). In contrast, ethylene glycol at 15% concentration performed the worst, with a sperm survival rate of only 5.31 ± 0.84% (Figure 1B). Therefore, dimethyl sulfoxide (DMSO) is considered the most suitable cryoprotectant for freezing and preserving *P. monodon* sperm, while glycerol is the second best choice.

### 3.3. The Effect of Different Equilibration Times at 4 °C

**Figure 2:**
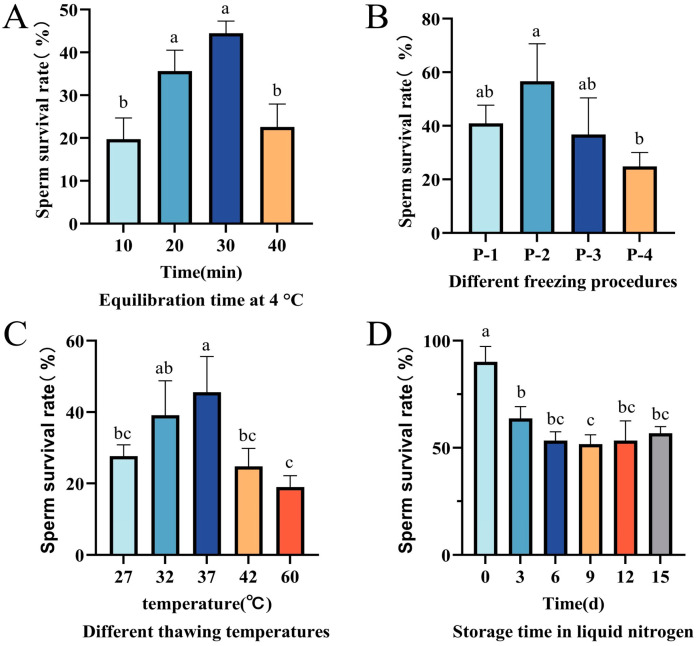
Post-cryopreservation sperm survival rates of *P. monodon* at different equilibration times (**A**), under different freezing protocols (**B**), thawing conditions (**C**), and cryopreservation durations (**D**). Different letters indicate significant differences between groups (*p* < 0.05).

The post-cryopreservation survival rate of *P. monodon* sperm varied significantly depending on the equilibration time. As shown in Figure 2A, the best results were obtained with an equilibration time of 30 min at 4 °C, yielding a significantly higher sperm survival rate (44.49 ± 2.83%) compared to both the 10-min and 40-min groups (*p* < 0.05). Conversely, the experimental group that was directly cryopreserved in liquid nitrogen after only 10 min of equilibration showed the worst results, with a sperm survival rate of just 19.71 ± 4.97% (Figure 2A). These findings suggest that both excessively short and excessively long equilibration times can negatively impact sperm survival rates, with 30 min being the optimal equilibration time.

### 3.4. Suitable Cooling Programs on Cryopreservation

The selection of the cryopreservation protocol played a crucial role in determining the survival rate of *P. monodon* sperm. The results indicated that protocol P-2 achieved the best outcomes, with a sperm survival rate of 56.65 ± 13.95% (Figure 2B). On the other hand, protocol P-4 performed the worst, yielding a survival rate of only 24.83 ± 5.21%. Notably, the P-2 group had a significantly higher survival rate compared to the P-4 group (*p* < 0.05). Therefore, it can be concluded that protocol P-2 is the most suitable method for the cryopreservation of *P. monodon* sperm.

### 3.5. Suitable Thawing Temperatures

As shown in Figure 2C, different thawing temperatures had a significant effect on the survival rate of frozen sperm (*p* < 0.05). The experimental results showed that when thawing at 37 °C in a water bath for 2 min, the sperm survival rate peaked (45.59 ± 9.99%), significantly higher than the other temperature groups (*p* < 0.05). The survival rate for thawing at 32 °C in a water bath for 2.5 min was 39.11 ± 9.67%, with no significant difference compared to the 37 °C group (*p* > 0.05) but significantly higher than the low and high temperature groups. The survival rates for thawing at 27 °C (4 min) and 42 °C (1.5 min) in a water bath were 27.65 ± 3.16% and 24.75 ± 5.08%, respectively. Thawing at 60 °C in a water bath (45 s) resulted in the lowest survival rate (18.98 ± 3.21%) due to heat shock causing more severe cell structure damage. The thawing times mentioned above were determined through preliminary experiments, representing the shortest time required for the complete thawing of liquid nitrogen-preserved samples (with the disappearance of ice crystals as the endpoint). Considering both efficiency and cell protection, 37 °C water bath thawing for 2 min was selected as the standard condition. This parameter balances the dual needs of rapid rewarming to reduce recrystallization damage and preventing high-temperature-induced membrane protein denaturation, providing a reliable basis for subsequent experiments.

### 3.6. Suitable Freezing Durations on Cryopreservation

Under identical cryopreservation conditions, the sperm survival rate was assessed over a 15-day storage period. The results revealed a notable decrease in the sperm survival rate within the first 0–3 days after cryopreservation. However, from days 3 to 15, the survival rate stabilized at around 50%, with no significant difference observed (*p* > 0.05) (Figure 2D).

### 3.7. Results of Sperm Morphology and Ultrastructure Analysis

#### 3.7.1. Scanning Electron Microscopy

**Figure 3:**
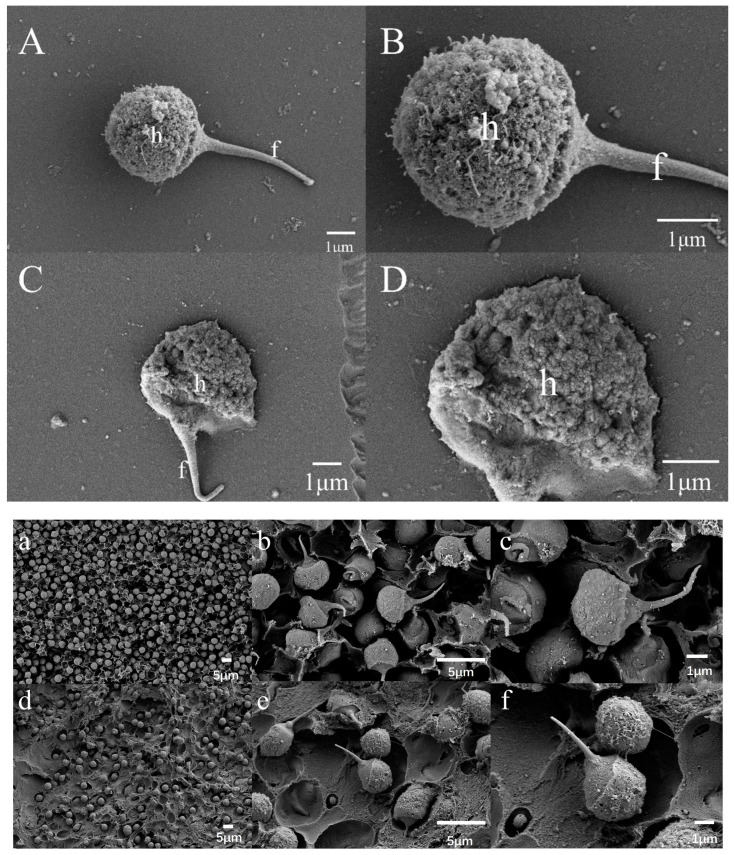
Scanning electron micrographs (SEM) of *P. monodon* sperm. (**A**,**B**) show sperm before cryopreservation, and (**C**,**D**) show sperm after cryopreservation. For quantitative analysis, images (**a**–**c**) depict sperm before cryopreservation, and (**d**–**f**) depict sperm after cryopreservation. h: head; f: flagellum.

Scanning electron microscopy observations revealed minor morphological differences in *P. monodon* sperm before and after freezing. Before freezing, the ultrastructure of the sperm cells appeared sound: the body of the sperm cells, acrosomal processes, and the outer layer of the cells had a slightly irregular surface but were undamaged (Figure 3A,B). After freezing, the overall morphology of the sperm cells remained largely unaffected, and the acrosomal processes were well-preserved. Although there was a slight increase in surface roughness, no obvious structural damage was observed (Figure 3C,D). Quantitative data showed that, based on scanning electron microscopy (SEM) measurements, the sperm spike length before freezing was 4.481 ± 0.03 μm, and the sperm width was 4.315 ± 0.09 μm. After freezing, the sperm spike length was 2.718 ± 0.08 μm, and the sperm width was 4.320 ± 0.05 μm. These findings suggest that the freezing treatment has minimal influence on sperm cell morphology and can effectively preserve its form.

#### 3.7.2. Transmission Electron Microscopy

**Figure 4:**
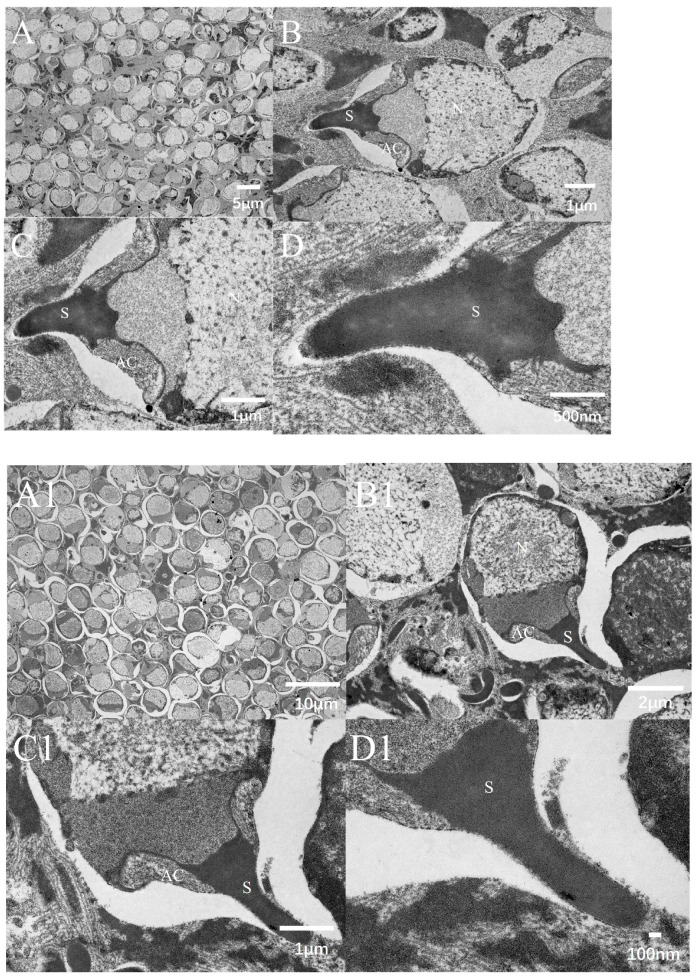
Transmission electron micrographs (TEM) of *P. monodon* sperm. The top row (**A**,**B**,**C**,**D**) depicts the TEM structure of sperm before freezing, while the bottom row (**A1**,**B1**,**C1**,**D1**) illustrates the TEM structure of sperm after freezing and thawing. (**A**,**A1**) show the general morphology of sperm; (**B**,**B1**) highlight the acrosome (AC), spike (S), and nucleus (N); (**C**,**C1**) zoom in to detail the acrosome and spike; and (**D**,**D1**) provide close-up views of the spike.S: spike; AC: acrosome; N: nucleus.3.8. Antioxidant Enzymes.

Transmission electron microscopy (TEM) images revealed distinct differences in the ultrastructure of *P. monodon* sperm between pre-freezing and post-freezing conditions. In the pre-freezing specimens (Figure 4), the acrosomal processes exhibited complete structural integrity with well-defined boundaries. The nuclear–subacrosomal interface was clearly demarcated, and no significant structural abnormalities were observed. The cytoplasm displayed uniform granularity without aggregation, and all organelles maintained their characteristic morphology, indicating that the sperm retained optimal physiological status at this stage. In contrast, post-freezing sperm cells (Figure 4) showed marked ultrastructural alterations. The acrosomal processes underwent partial detachment accompanied by morphological deformation, while the acrosome appeared compressed with reduced electron density compared to pre-freezing conditions. Freezing-induced damage manifested as blurring of the nuclear–subacrosomal boundary. Numerous irregular vesicles were frequently observed within the cytoplasm, suggesting cryo-injury-induced redistribution or degradation of intracellular components. Quantitative analysis demonstrated significant dimensional changes: sperm spike length decreased from 2.368 ± 0.04 μm (pre-freezing) to 2.074 ± 0.07 μm (post-freezing), and sperm width was reduced from 4.180 ± 0.06 μm to 3.786 ± 0.02 μm. These findings collectively indicated that cryopreservation compromised sperm structural integrity through multiple mechanisms.

**Figure 5:**
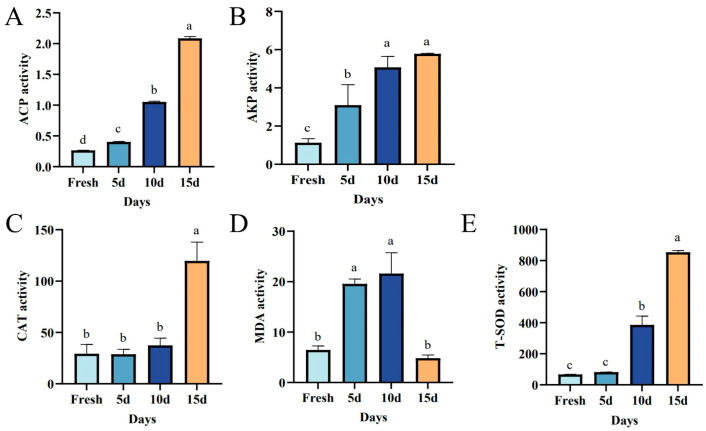
Effects of different freezing durations on antioxidant enzyme activities of *P. monodon* sperm. Panel (**A**) shows the ACP activity, panel (**B**) depicts the AKP activity, panel (**C**) shows the CAT activity, panel (**D**) shows MDA activity, and panel (**E**) shows T-SOD activity. Different letters represent significant differences between groups (*p* < 0.05).

The enzyme activities of AKP and ACP in frozen sperm of *P. monodon* gradually increased as freezing time extended, showing significant differences (*p* < 0.05). Initially, the activity of MDA rose, but then it decreased with prolonged freezing time. As for CAT enzyme activity, there were no significant differences from 0 to 10 days (*p* > 0.05); however, a noticeable upward trend emerged at 15 days (*p* < 0.05). Similarly, T-SOD did not show significant differences from 0 to 5 days (*p* > 0.05), but it exhibited an upward trend from 10 to 15 days (*p* < 0.05) (Figure 5).

## 4. Discussion

### 4.1. Effects of Dilutors on Sperm Survival Rate

In crustacean sperm cryopreservation, selecting an appropriate diluent is essential for maintaining sperm viability and function. Diluent solutions are critical in regulating osmotic pressure, pH, and ionic composition. Natural seawater and calcium-free artificial seawater have been widely used in crustacean sperm cryopreservation studies [8,25] Bray and Lawrence found that both seawater and calcium-free artificial seawater can serve as effective buffers for sperm morphological evaluation [26]. In studies on the acrosome reaction of *Portunus trituberculatus* sperm, Wang et al. found that Ca^2+^-FSW was the optimal sperm buffer [27].

In this study, we evaluated the cryopreservation effectiveness of four different diluents—NSW, RS, HBSS, and D-Hanks—on *P. monodon* sperm. The osmolarities of each diluent were as follows: NSW was 1020 ± 15 mOsm/L, RS was 305 ± 3 mOsm/L, HBSS was 290 ± 2 mOsm/L, and D-Hanks was 295 ± 4 mOsm/L. Osmotic pressure and ionic composition are key factors affecting sperm viability. Our experimental results demonstrate that natural seawater (NSW) provides the best protection during cryopreservation, with post-thaw sperm viability significantly higher than that observed with other diluents (Figure 1). This phenomenon may be attributed to its osmotic pressure and ionic composition, which closely mimic the physiological state of prawn sperm in their natural environment. The salinity, pH, and abundance of trace elements (such as calcium and magnesium) in natural seawater likely act synergistically to maintain sperm membrane integrity and metabolic activity. These findings are consistent with previous studies. This result is consistent with the study by Chen et al. [28], who showed that Na^+^ and K^+^ ions play a crucial role in maintaining sperm vitality. Similarly, in their cryopreservation study of sea cucumber (*Apostichopus japonicus*) sperm, Xu et al. [29] observed that filtered natural seawater combined with an optimized cryoprotectant significantly improved post-thaw sperm viability (exceeding 65%). They suggested that the complex ionic environment of natural seawater helps reduce stress-induced damage during the freezing process. In contrast, although Ringer’s solution differs in ionic composition from natural seawater, its balanced design in terms of osmotic pressure and key ions (e.g., Na^+^, K^+^, Ca^2+^) results in relatively good preservation effects, ranking only slightly behind natural seawater in our experiments. However, HBSS and D-Hanks performed poorly, with significantly lower post-thaw sperm viability. This may be due to their ionic composition and osmotic pressure not fully matching the cryopreservation requirements for prawn sperm. Le et al. [30] also found in their study on the sperm of striped Jewfish (*Stereolepis doederleini*) that osmotic pressures below or above 35 PSU lead to reduced sperm viability, indicating that deviations from the natural osmotic state may cause irreversible damage to sperm. Additionally, Chang et al. [31] demonstrated in their cryopreservation study of *Portunus trituberculatus* sperm that the use of Ca^2+^-free artificial seawater as a diluent significantly reduced both sperm viability and enzyme activity, further supporting the importance of ionic components (such as Ca^2+^) in maintaining sperm functionality. The efficacy of different diluents has also been confirmed in other aquaculture studies. For example, studies have shown that using calcium-free artificial seawater with 20% glycerol significantly improves the sperm survival rate of *P. trituberculatus*, similar to optimized natural seawater and calcium balance [31]. Likewise, research on *Sebastes schlegelii* sperm cryopreservation demonstrated that even under optimized diluent conditions, the freezing process still affects sperm DNA integrity and mitochondrial activity, underscoring the cellular damage caused by cryopreservation. Transcriptome and methylome analyses in this study revealed the impact of cryopreservation on the expression of multiple genes, further emphasizing the need to optimize diluent composition to minimize cellular damage [32]. These differences in cryoprotective effects highlight the significant impact of diluent composition, including calcium, sodium, and potassium ion concentrations, on sperm viability and survival rates.

Our study demonstrates that osmotic pressure and ionic equilibrium of the diluent should be prioritized as critical factors when selecting extenders for sperm cryopreservation in *P. monodon*. Natural seawater, owing to its chemical properties that closely resemble the species’ natural habitat, has demonstrated the most superior performance in maintaining sperm motility and cryopreservation efficacy. Nevertheless, considering the regional variability in composition of natural seawater, future investigations should focus on optimizing artificial seawater formulations (e.g., through adjustment of Na^+^, K^+^, and Ca^2+^ concentrations or supplementation with trace elements) to achieve the dual objectives of standardization and efficient cryopreservation.

### 4.2. Effects of Cryoprotectants on Sperm Survival Rate

Due to its excellent permeability and cryoprotective properties, dimethyl sulfoxide (DMSO) has been extensively employed in cryopreservation protocols for crustacean spermatozoa, with applications typically utilizing the minimum effective concentration to mitigate its inherent cytotoxic effects, including *Sicyonia ingentis* [8], *Scylla serrata* [22], *Penaeus monodon* [14,15], and *Litopenaeus vannamei* [16].

Previous studies have demonstrated that 10% DMSO as a standard cryoprotectant concentration achieves sperm survival rates of 38–56% in the cryopreservation of *P. monodon* sperm [14,17]. In the present study, a significantly enhanced survival rate of 60.49% was observed when using 10% DMSO, with this improvement potentially attributable to three critical technical modifications: (1) the implementation of natural seawater (30ppt salinity) as the diluent instead of the artificial seawater conventionally utilized in prior literature; (2) the development of a stepwise cooling protocol (4 °C → −20 °C → −80 °C → liquid nitrogen with 5 min holds at each transitional temperature stage), contrasting with the conventional practice of direct liquid nitrogen immersion; and (3) the optimization of pre-cooling equilibration duration to 30 min at 4 °C prior to freezing initiation, effectively balancing osmotic efficiency with reduced cryoprotectant toxicity exposure.

The synergistic interaction between the cooling rate and the composition of the carrier solution may represent a critical determinant. Szurek et al. [33] further conducted a systematic comparison of the toxicity of DMSO, ethylene glycol, and propylene glycol, noting that DMSO has low toxicity at commonly used concentrations (such as 5–10%), with relatively minimal negative effects on cell survival rate, fertilization rate, and development, whereas propylene glycol showed higher toxicity. This property makes DMSO particularly suitable for cell types that are sensitive to toxicity during cryopreservation, such as sperm and oocytes. Therefore, DMSO has significant advantages as the preferred cryoprotectant for sperm cryopreservation. Claudet et al. [11] found that combining 10% DMSO and 10% PG significantly enhanced sperm viability in *Macrobrachium rosenbergii*, surpassing the effects of single cryoprotectants like DMSO, PG, and glycerol, suggesting that combined cryoprotectants may further improve sperm survival rates.

Furthermore, the optimal concentration of DMSO exhibits a close correlation with sperm morphological characteristics. Anchordoguy et al. [8] observed that 5% DMSO was optimal for *Sicyonia ingentis*, while Ke and Cai [13] recommended a combination of 10% DMSO and 5–10% glycerol for *P. chinensis*, indicating a high degree of species dependency in cryoprotectant choice and concentration among crustaceans. Future studies could further explore the combined use of DMSO with other cryoprotectants to optimize cryopreservation outcomes.

### 4.3. The Effect of Equilibration Time on Sperm Survival Rate

In sperm cryopreservation, equilibration time is crucial for the penetration of cryoprotectants and for sperm adaptation to low temperatures. This study shows that the optimal equilibration time for *P. monodon* sperm is 30 min, which provides the best cryoprotective effect; both shorter and longer equilibration times lead to reduced survival rates. Regarding the optimization effect of equilibration time on cryoprotectant permeation, multiple pieces of evidence have been accumulated in related crustacean studies. For example, in the study of Japanese marsh shrimp (*Macrobrachium nipponense*), sperm resting at 4 °C for 30 min significantly improved survival rates, which the researchers attributed to the improvement in cryoprotectant permeation efficiency [34]. Similarly, Liu et al. [35] observed in their experiment on Japanese crab (*Charybdis japonica*) that a 20-min equilibration time achieved a balance between cryoprotectant absorption and toxicity damage. These studies collectively suggest that the specific equilibration time must be adjusted according to the biological characteristics of the species, but the core mechanism points to optimizing the interaction between the cryoprotectant and the cells through time control.

Similarly, Liu et al. showed that the optimal equilibration time for *C. japonica* sperm was 20 min, with both longer and shorter times reducing sperm viability, possibly due to metabolic depletion or the accumulation of cryoprotectant toxicity [35]. Ke and Cai found that an equilibration time of 20–30 min at 0–4 °C effectively prevented the acrosome reaction in *P. chinensis* sperm, further validating the importance of equilibration time in cryoprotection [13].

Other studies have shown that equilibration time should be adjusted according to the type of cryoprotectant and species-specific characteristics. For example, Nimrat et al. found that a shorter equilibration time (e.g., 20 min) with low-concentration DMSO significantly reduced cryodamage in *Fenneropenaeus merguiensis* sperm, thereby improving sperm survival rates [36]. This suggests that a shorter equilibration time helps prevent the accumulation of cryoprotectant toxicity, thus preserving sperm viability. In contrast, Vuthiphandchai et al. observed that extending the equilibration time to 60 min or more significantly decreased sperm viability, indicating that prolonged equilibration may lead to the buildup of cryoprotectant toxicity, detrimental to sperm survival [15]. This observation is consistent with our experiment, where longer equilibration times led to decreased survival rates in *P. monodon* sperm, further emphasizing the critical balance required in equilibration time to ensure cryoprotectants provide protection without excessive toxicity.

Additionally, Chang et al. found that 15 min was the optimal equilibration time for *P. trituberculatus*, suggesting that a short equilibration time allows effective cryoprotectant penetration while avoiding oxidative stress or metabolic consumption associated with longer times [31]. This is consistent with Gwo and Lin’s finding that prolonged equilibration time led to decreased embryo survival rates in *Penaeus japonicus*. Prolonged equilibration may cause sperm to metabolize continuously at low temperatures, thereby increasing oxidative stress and leading to cellular damage [37]. Therefore, while cryoprotectants provide effective protection over short equilibration times, extended equilibration may induce additional stress responses, negatively impacting sperm viability.

### 4.4. Effects of Cooling Protocol and Thawing Conditions on Sperm Survival Rate

In sperm cryopreservation, the cooling rate and thawing temperature significantly impact cell survival. Using a programmable cooling device, we employed a slow freezing method, cooling *P. monodon* sperm at a rate of −5 °C/min down to −20 °C, equilibrating, and then gradually lowering the temperature to −180 °C, where it was stored in liquid nitrogen, achieving optimal cryoprotection. This stepwise cooling process facilitated the gradual formation of extracellular ice crystals, effectively reducing the risk of intracellular ice crystal formation and minimizing cell dehydration and morphological deformation due to osmotic pressure changes. This is consistent with the theory proposed by Woods et al., which states that slow freezing improves cell survival rate [38,39].

In contrast, Vuthiphandchai et al. found that a freezing rate of −2 °C/min achieved higher survival rates for *P. monodon* spermatophores, whereas faster rates led to complete sperm death [15]. This difference may be due to the gel matrix layer in the spermatophore structure, which affects cryoprotectant penetration and thus creates different cryopreservation requirements between spermatophores and individual sperm cells.

The choice of thawing temperature is equally crucial. Studies have shown that sperm frozen at optimal cooling rates are susceptible to damage during thawing if an appropriate thawing method is not used [5]. Chang et al. reported that thawing *P. trituberculatus* sperm in a 37 °C water bath yielded the best results, consistent with Yang et al.’s findings on *Litopenaeus vannamei* [31,40]. This may be because the 37 °C temperature helped maintain cell membrane integrity during rapid thawing, avoiding further damage from higher temperatures. Studies indicate that the optimal thawing temperature for *P. chinensis* sperm is 35–40 °C [13], while for *P. trituberculatus* sperm, it is 55 °C [5], highlighting that different species have specific thawing temperature requirements. This suggests that coordinating the cooling and thawing processes in cryopreservation is essential to minimize cell damage and improve survival rates. Future research could further investigate the interactions between cooling and thawing methods to optimize cryopreservation techniques for sperm and other sensitive cells.

### 4.5. Effects of Cryopreservation on the Ultrastructure of Sperm in P. monodon

Sperm morphological characteristics hold significant taxonomic value in crustacean species identification, particularly within the Penaeidae family, where the evolutionary specialization of sperm structures reflects adaptive differentiation in reproductive strategies. The sperm of *P. monodon* exhibits a characteristic acrosomal spike complex, comprising three distinct regions: the head region (nuclear compartment), the acrosomal vesicle (enzyme storage domain), and a non-motile acrosomal spike. In this study, we employed scanning electron microscopy (SEM) and transmission electron microscopy (TEM) to examine the ultrastructure of *P. monodon* sperm, revealing the structural changes that occur before and after cryopreservation.

Prior to freezing, *P. monodon* sperm exhibited a characteristic non-motile acrosomal spike complex. The sperm head appeared robust, with a clearly visible and structurally intact acrosomal spike showing no signs of damage. Transmission electron microscopy (TEM) revealed a well-defined boundary between the nucleus and subacrosomal region, evenly distributed cytoplasm, and well-preserved organelles, collectively indicating optimal physiological integrity at this stage. Following cryopreservation, scanning electron microscopy (SEM) observations showed that while the overall external morphology of the sperm remained relatively intact, the surface of the acrosomal spike appeared slightly roughened. Notably, no significant rupture or structural damage was detected, and the acrosomal spike remained largely intact without apparent fragmentation or detachment.

However, TEM analysis revealed internal structural changes after freezing, including vacuolation and granulation in the cytoplasm. This could be attributed to incomplete elimination of intracellular water during the freezing process, leading to ice crystal formation. This also resulted in a loss of distinction between the nucleus and subacrosome, indicating that mechanical stress during freezing had an irreversible impact on the internal sperm structure.

Similar effects of cryopreservation on sperm ultrastructure have been observed in other species. For example, studies on rainbow trout (*Oncorhynchus mykiss)* sperm [41] reported membrane rupture, flagellar breakage, and organelle damage due to freezing, which are consistent with the damage patterns seen in *P. monodon* sperm in this study. In African turquoise killifish (*Nothobranchius furzeri*) sperm cryopreserved under certain conditions [42] it was found that after freeze–thaw, the sperm motility was poor, although the structural shape remained fairly good.

When DMSO was used as a cryoprotectant with appropriate freezing and thawing rates, the sperm mitochondria and flagellum of *P. monodon* remained largely intact, similar to the mitochondrial stability observed in other species’ cryopreserved sperm. This indicates that sperm tolerance to cryopreservation varies significantly among species. For instance, research on blue mussel (*Mytilus galloprovincialis*) [43] showed that different cooling rates significantly affected sperm activation rate and ultrastructural damage, which aligns with the damage patterns observed in *P. monodon* sperm in this study. However, it appears that the currently selected preservation temperatures do not prevent ultrastructural sperm damage, as internal damage, such as vacuole formation and loss of mitochondria, still occurs during cryopreservation.

Furthermore, the structural responses of sperm to cryopreservation differ across species. The external morphological damage to cryopreserved *P. monodon* sperm was relatively minor, with the flagellum and acrosomal spike on the head remaining intact. Compared to some teleost fish, such as ayu, *P. monodon* sperm demonstrates greater tolerance to freezing, particularly in protecting external structures.

It is noteworthy that partial acrosomal spikes exhibited characteristic curling after cryopreservation, with morphological alterations closely resembling the early-stage acrosome reaction patterns reported by Pongtippatee et al. [44] in *P. monodon*. This structural observation, coupled with the relatively high viability rate (82.3 ± 3.1%) demonstrated by trypan blue exclusion assay, suggests potential retention of partial functional activity in post-thaw spermatozoa. However, given the absence of direct functional assessments and in Vitro acrosome reaction induction experiments in the current investigation, it remains undetermined whether these ultrastructural modifications represent biologically significant changes or merely cryo-induced physical deformations. Subsequent studies should incorporate functional assays to elucidate the actual impacts of cryopreservation on acrosomal reactivity and comprehensive functional competence of sperm cells.

### 4.6. Effects of Cryopreservation on the Antioxidant Function of Sperm in P. monodon

T-SOD and CAT are crucial antioxidant enzymes in sperm, playing pivotal roles in neutralizing reactive oxygen species (ROS) and shielding cells from oxidative damage. Their activities are closely tied to the overall antioxidant enzyme activity of organisms or cells, and fluctuations in this activity can serve as an indicator of stress or damage inflicted by external environmental factors [45,46].

In our study, all five sperm enzymes exhibited an increase, with the exception of MDA. These results are consistent with the majority of published studies on the impact of cryopreservation on sperm enzyme activity [45]. This rise may be attributed to mechanical damage during the freezing and thawing process, which compromises the cell membrane structure, leading to the leakage of enzymes within the sperm [46].

Notably, the activities of T-SOD, AKP, and ACP in sperm significantly increased after cryopreservation, while CAT activity remained relatively stable from 0 to 10 days. On one hand, this suggests that the cryopreservation technique used in this study effectively protected *P. monodon* sperm, as the internal enzyme contents were well-preserved without significant leakage. On the other hand, it also indicates that sperm cells experienced environmental stress during cryopreservation, which may have impacted energy metabolism and ROS scavenging processes essential for surviving adverse conditions.

Interestingly, Kankofer et al. reported a decrease in SOD and CAT activities in cryopreserved horse (*Equus caballus*) sperm [47], which contrasts with our findings. This discrepancy may be attributed to various factors affecting antioxidant enzyme activity during the sperm preservation process, such as freezing stress, membrane damage, oxidative stress-related responses, and cell-native adaptation mechanisms. These changes underscore that cells undergo numerous physiological and biochemical stressors during cryopreservation, and different species may respond differently to these stressors.

## 5. Conclusions

In this study, we refined the cryopreservation protocol for *P. monodon* sperm. We utilized natural seawater as a diluent and incorporated 10% dimethyl sulfoxide (DMSO) as a cryoprotectant. The optimal conditions for standing, cooling, and thawing were meticulously determined: a 30-min standing period at 4 °C, followed by a cooling program of −5 °C/min to −20 °C with a 5-min hold, then −10 °C/min to −80 °C with another 5-min hold, and ultimately −20 °C/min to −180 °C for storage in liquid nitrogen. Sperm samples that underwent this cryopreservation process and were stored in liquid nitrogen for 15 days exhibited high vitality. Scanning electron microscopy (SEM) confirmed that the sperm cells sustained minimal damage both before and after freezing, thanks to the optimized cryopreservation procedure, which had minimal impact on the overall cell structure. However, transmission electron microscopy (TEM) observations revealed some internal damage to the sperm cells post-freezing. This indicates that while cell viability remained high, changes in the ultrastructure still warrant attention during the cryopreservation process. Furthermore, enzyme activity assays suggested that the frozen sperm possessed the high biological activity necessary for fertilization. These results provide valuable insights for the conservation of *P. monodon* genetic resources, streamline the breeding of parent stocks in aquaculture, and address the challenge of asynchronous sexual maturation between male and female parents.

In subsequent studies, we will investigate the use of two or more cryoprotectants to further enhance the cryopreservation formula and, consequently, improve sperm survival rates.

## Figures and Tables

**Table 1:** Cooling protocols for cryopreservation of *Penaeus monodon* sperm using a programmable cryocooler.

Serial Number	Cooling Program
P-1	−5 °C/min to −80 °C; hold at −80 °C for 5 min; −20 °C/min to −180 °C; stored in liquid nitrogen
P-2	−5 °C/min to −20 °C; hold at −20 °C for 5 min; −10 °C/min to −80 °C; hold at −80 °C for 5 min; −20 °C/min to −180 °C; stored in liquid nitrogen
P-3	−5 °C/min to −20 °C; hold at −20 °C for 5 min; −20 °C/min to −80 °C; hold at −80 °C for 5 min; stored in liquid nitrogen
P-4	−5 °C/min to −80 °C; stored in liquid nitrogen

## Data Availability

The raw data used to support the findings of this study are available from the corresponding author upon request: T.L. and F.Z., zhoufalin0925@163.com, ltm600@163.com.
